# Genomic epidemiology and antimicrobial susceptibility of clinical *Mycoplasma pneumoniae* isolates in Shanghai, 2023–2024

**DOI:** 10.3389/fcimb.2026.1747629

**Published:** 2026-07-01

**Authors:** Qianyue Wu, Fen Pan, Na Wang, Fangyuan Yu, Tiandong Zhang, Huihong Qin, Yingying Shi, Xiaozhou Pan, Wenhao Weng, Dingding Han, Hong Zhang

**Affiliations:** 1 Department of Clinical Laboratory, Shanghai Children’s Hospital, School of Medicine, Shanghai Jiao Tong University, Shanghai, China; 2 Institute of Pediatric Infection, Immunity, and Critical Care Medicine, Shanghai Children’s Hospital, School of Medicine, Shanghai Jiao Tong University, Shanghai, China; 3 Department of Pulmonary and Critical Care Medicine, Shanghai Huadong Hospital, Fudan University, Shanghai, China

**Keywords:** macrolide resistance, molecular epidemiology, *Mycoplasma pneumoniae*, severe *Mycoplasma pneumoniae* pneumonia, whole-genome sequencing

## Abstract

**Introduction:**

*Mycoplasma pneumoniae* (*M. pneumoniae*) is a leading cause of respiratory infection in school-age children. A global resurgence occurred in 2023-2024, yet whole-genome molecular epidemiology and antimicrobial susceptibility data remains limited.

**Methods:**

In this study, we isolated *M. pneumoniae* strains from children with respiratory infections in Shanghai, collected clinical record data, and profiled antibiotic susceptibility using the broth microdilution method. Whole-genome sequencing was performed on all isolates and globally available *M. pneumoniae* genomes from GenBank were incorporated for comparative analysis.

**Results:**

A total of 148 *M. pneumoniae* strains were isolated, among which 79.7% (118/148) were from patients diagnosed with severe *M. pneumoniae* pneumonia (SMPP). All isolates were highly resistant to macrolides but remained susceptible to tetracyclines and fluoroquinolones. Of note, P1-2-type strains showed lower resistance levels to azithromycin and 16-membered macrolides compared with P1-1. Although the predominant genotypes were still P1-1 (92.6%), ST3 (88.5%), and M4-5-7-2 (79.7%), several previously uncommon MLVA types—M3-5-7-2, M4-3-7-2, and M4-4-7-2—have increased in frequency since the COVID-19 pandemic and were associated with milder inflammatory responses. Genomic analysis revealed high conservation across strains. Notably, a higher number of *hsdS* gene homologs, which serve as epigenetic modulators, correlated with reduced SMPP incidence and lower CD3^+^/CD4^+^ T-cell counts.

**Discussion:**

In this study we provided the first comprehensive WGS analysis and updated MIC profiles of clinically isolated *M. pneumoniae* in Shanghai. Our findings highlight the persistent macrolide resistance and the emergence of genomic variations that warrant ongoing surveillance. High virulence carriage and high macrolides resistance may be among the factors contributing to elevated SMPP levels.

## 1 Introduction

As a common respiratory pathogen, *Mycoplasma pneumoniae* (*M. pneumoniae*) accounts for approximately 20%–40% of community-acquired pneumonia cases in pediatric populations. It predominantly infects school-age children aged 5–15 years (Waites et al., 2017). Periodic outbreaks of *M. pneumoniae* infections occurs every 3–7 years, with each outbreak lasting 1–2 years (Jacobs, 2012). The most recent epidemic of *M. pneumoniae* emerged in May 2023 and persisted until 2024, affecting countries across Asia, Europe and America (Sauteur et al., 2025).

*M. pneumoniae* primarily attaches to airway epithelial cells through surface cell-adhesion molecules on its membrane. These molecules are composed of a variety of adhesion proteins that work in concert to facilitate attachment (He et al., 2016). Most infected children exhibit persistent fever and cough, and these symptoms are generally self-resolving (Waites et al., 2017). Nevertheless, some patients may progress to severe *M. pneumoniae* pneumonia (SMPP), which is characterized by persistent high fever, progressive clinical deterioration, extensive lobar consolidation, and extrapulmonary complications such as encephalitis and rash (Sánchez-Vargas and Gómez-Duarte, 2008; Liew et al., 2022). Research has demonstrated that during the 2023 *M. pneumoniae* pandemic, the prevalence of SMPP among pediatric populations surpassed previous outbreaks, exceeding 50% (Wu et al., 2024).

Macrolide antibiotics serve as the first-line treatment for *M. pneumoniae* infections. However, macrolide-resistant *M. pneumoniae* (MRMP) infections have become increasingly prevalent in China, posing significant treatment challenges. Macrolide resistance arises from mutations in the 23S rRNA or ribosomal proteins L4 and L22 of *M. pneumoniae*, with A2063G mutation being the most common. In the face of MRMP, pediatricians recommend alternative antibiotics such as tetracyclines and fluoroquinolones (Pereyre et al., 2016). However, tetracyclines are only advised for children aged 8 years and above due to the risk of dental staining and enamel hypoplasia, while fluoroquinolones are used cautiously in individuals under 18 years of age due to concern about potential musculoskeletal damage. Owing to its slow growth rate and stringent culture conditions, successfully culturing *M. pneumoniae in vitro* is challenging, which has consequently led to a relative scarcity of antimicrobial susceptibility assay for this pathogen. In the Shanghai region, the available antimicrobial susceptibility data must be tracked back five years. Given that long-term use of antibiotics may induce drug resistance, continuous monitoring of antibiotics resistance phenotypes is of great significance.

Multiple genotyping methods have been employed for *M. pneumoniae*, including P1 typing (Spuesens et al., 2009), multi-locus variable number tandem repeat analysis (MLVA) (Dégrange et al., 2009) and multi-locus sequence typing (MLST) (Brown et al., 2015). Studies have revealed characteristic differences among various *M. pneumoniae* types. For example, it was reported that the prevalence of MRMP of P1–1 strain, defined by 23S rRNA mutation, was higher than that of P1–2 strains before the COVID-19 pandemic (Wang et al., 2022). However, after the pandemic, there had been a dramatic surge in the MRMP rates of P1–2 strains (Li et al., 2024). The ST3 lineage has demonstrated an increased frequency of homologous recombination events and greater clonal diversity (Hsieh et al., 2022). Moreover, a recent report identified M4-5-7–2 genotype as having enhanced virulence factors than M3-5-6-2, which was considered to be a driving force behind the *M. pneumoniae* outbreak in Beijing following the COVID-19 pandemic (Jiao et al., 2025). Given these findings, molecular typing of *M. pneumoniae* on the whole genome level not only aids in understanding the epidemiological characteristics and transmission patterns of the pathogen, but also provides crucial insights for the development of targeted prevention and treatment strategies.

Surprisingly, as a prominent metropolitan center, Shanghai has previously been devoid of comprehensive whole genome analysis and detailed antimicrobial susceptibility profiles for *M. pneumoniae*. To bridge this knowledge gap, we conducted a multi-faced approach, performing whole-genome sequencing, clinical data analysis, and antimicrobial susceptibility testing on 148 *M. pneumoniae* isolates collected from Shanghai in 2023-2024. Furthermore, we integrated 449 *M. pneumoniae* sequences from the GenBank database to investigate the phylogenetic evolution and global distribution patterns of *M. pneumoniae*.

## 2 Materials and methods

### 2.1 Sample collection and *M. pneumoniae* culture

To investigate the prevalence of *M. pneumoniae*, we compiled the results of all pathogen PCR tests conducted in our hospital from December 2023 to December 2024. These cases were suspected respiratory tract infections in children, diagnosed and referred for testing by physicians. To investigate the genomic characteristics of *M. pneumoniae* during the same period, we continuously collected strains from *M. pneumoniae* culture-positive specimens in the clinical laboratory, independent of the pathogen PCR assay. Then, *M. pneumoniae* strains were isolated and re-cultured in mycoplasma broth containing mycoplasma broth base CM403 (Oxoid), mycoplasma selective supplement G SR59 (Oxoid), 0.5% glucose, and 0.002% phenol red at 37 °C (Sun et al., 2008; Wang et al., 2022). The *M. pneumoniae* strain M129 (ATCC 29342) was employed as a quality control reference. For specimens where the culture medium exhibited a yellow color change, further confirmation was carried out via PCR. We used primers (5’-GCCACCCTCGGGGGCAGTCAG-3’ and 5’-GAGTCGGGATTCCCCGCGGAGG-3’) to specially amplify a 209-bp fragment of the P1 gene (Wang et al., 2022).

### 2.2 Antimicrobial susceptibility test

Antimicrobial susceptibility testing was performed using the broth microdilution method. All experimental operations and result interpretations adhered strictly to the CLSI M43-A guidelines. Positive control wells, negative control wells, and antibiotic control wells were included. The microtiter plate was sealed and incubated at 37 °C until a color changed from red to yellow was observed in the positive control wells. The antibiotics tested included erythromycin, azithromycin, clindamycin, clarithromycin, josamycin, tetracycline, minocycline, doxycycline, levofloxacin, and moxifloxacin.

### 2.3 Whole-genome sequencing and genome annotation

DNA was extracted from 1.5mL positive culture medium using Mag Soil/Stool DNA Kit (Yeasen Biotech). The sequencing library was created using VAHTS Universal Plus DNA Library Prep Kit for Illumina V2 (Vazyme Biotech). Whole genome sequencing was carried out on the Illumina NovaSeq 6000 high-through-sequencing platform. Paired-end sequencing was used with a read length of 150 bp. The raw reads were filtered using the fastp (v0.23.4) to discard low-quality reads. The filtering criteria were as follow: (1) reads with more than 5% N-base content; (2) reads with over 50% bases of low quality (quality score ≤ 5); (3) reads contaminated with adapter sequences; and (4) PCR duplicate sequences resulting from amplification. After quality control, the reads were assembled using EToKi v1.3 to generate contigs. Gene prediction and annotation were subsequently conducted using Prokka v1.14.6.

### 2.4 Molecular typing

P1 genotypes were identified based on predefined single nucleotide variant (SNV) sites specific to the *M. pneumoniae* P1 gene. Specifically, the *M. pneumoniae* strain FH (NCBI accession number: GCA_900660465), representing the P1–2 genotype, served as the ancestral reference genome. For each sample, total read support for the ancestral allele (P1-2) and derived allele (P1-1) was calculated by summing the read depths across all predefined P1 SNV loci. When the read depth met the required threshold, if the support for the ancestral allele was higher than that of the derived allele, the sample was assigned as P1–2 type; otherwise, it was assigned as P1–1 type. The analysis pipeline is available at: https://github.com/yanghuajiang/MPtyper.

To determine the MLVA types, we aligned the assembled sequences of the strains against the primer sequences of four loci (Mpn13, Mpn14, Mpn15, and Mpn16) from the reference genome GCF_000027345 to extract the sequences of the target loci, and subsequently employed the TRF software (https://github.com/Benson-Genomics-Lab/TRF) to predict and quantify the tandem repeat sequences (Dégrange et al., 2009).

MLST was performed based on the sequences of eight housekeeping genes (*ppa, pgm, gyrB, gmk, glyA, atpA, arcC*, and *adk*) (Brown et al., 2015) according to the PubMLST scheme for *M. pneumoniae*, using MLST v2.23.0 (https://github.com/tseemann/mlst) to determine sequence types (STs).

### 2.5 Virulence gene analysis and macrolides resistance gene mutation detection

The coding sequences were aligned to the Virulence Factor Database (VFDB) (http://www.mgc.ac.cn/VFs/main.htm) to identify putative virulence genes. The alignment was performed with stringent criteria, requiring a query coverage of ≥50% and an identity of ≥90%.

### 2.6 Phylogenetic tree construction

Using the “align” module of EToKi v1.3, the selected 148 genomes and the genome sequences obtained from NCBI were aligned with the reference genome (GCF_000027345) to obtain a multiple sequence alignment matrix of non-repetitive core genomic regions shared by ≥95% of the genomes. Subsequently, the “EToKi phylo” module was used to perform maximum likelihood phylogenetic analysis on the obtained alignment results. Additionally, the RecHMM was employed to remove recombinant regions, thereby producing a maximum likelihood phylogenetic tree devoid of recombination. The Interactive Tree of Life v6.8 was applied for visualization and annotation of strain information.

### 2.7 OrthoFinder homology analysis software

The OrthoFinder v2.5.4 was used for gene family analysis to find potential homologous genes. The *M. pneumoniae* strain M129 (GCF_000027345) was utilized as a reference for homologous gene comparison. This analysis facilitated cluster comparison using the Markov Cluster Algorithm (MCL). Principal component analysis (PCA) and correlation analysis were performed based on the orthogroup count matrix using R v4.2.2 and visualization was conducted using ggplot2 package.

### 2.8 Gene variation analysis

Based on the assembled sequence, the EToKi v0.3 toolkit was employed to perform type annotation of variant sites using the reference sequence GCF_000027345 and its gene annotation information. Furthermore, macrolide resistance within 23S rRNA and the ribosomal proteins L4 and L22 were defined by mapping sequencing reads to the reference genome using minimap2. Alignment files were processed with SAMtools to extract base-level information at target loci, enabling the determination of resistance-associated variants. The detailed analysis workflow followed the pipeline: https://github.com/yanghuajiang/MPtyper.

### 2.9 Collection of clinical characteristics

Clinical data collected from patients, including demographic information, clinical presentations, laboratory findings, treatment courses, hospitalization records, and other relevant information. MPP and SMPP were diagnosed by professional pediatricians, adhering to “Guidelines for Diagnosis and Treatment of *Mycoplasma pneumoniae* Pneumonia in Children (2023 edition)” (Zhao et al., 2024). This study was approved by the Ethics Committee of the Children’s Hospital of Shanghai (2025R145-E01).

### 2.10 Statistical analysis

Statistical analyses were conducted using SPSS v27.0 to assess differences between groups. Categorical variables were expressed as percentages. For continuous variables, comparisons were using Student *t* test or Mann-Whitney *U* test. Categorical variables were compared using Chi-square test or Fisher’s exact test. A *P*-value of <0.05 was considered to indicate a statistically significant difference. Additional graphical analyses and visualizations were performed using R v4.2.2 or Python v3.8.

## 3 Results

### 3.1 Demographic statistics and clinical characteristics

**Figure 1:**
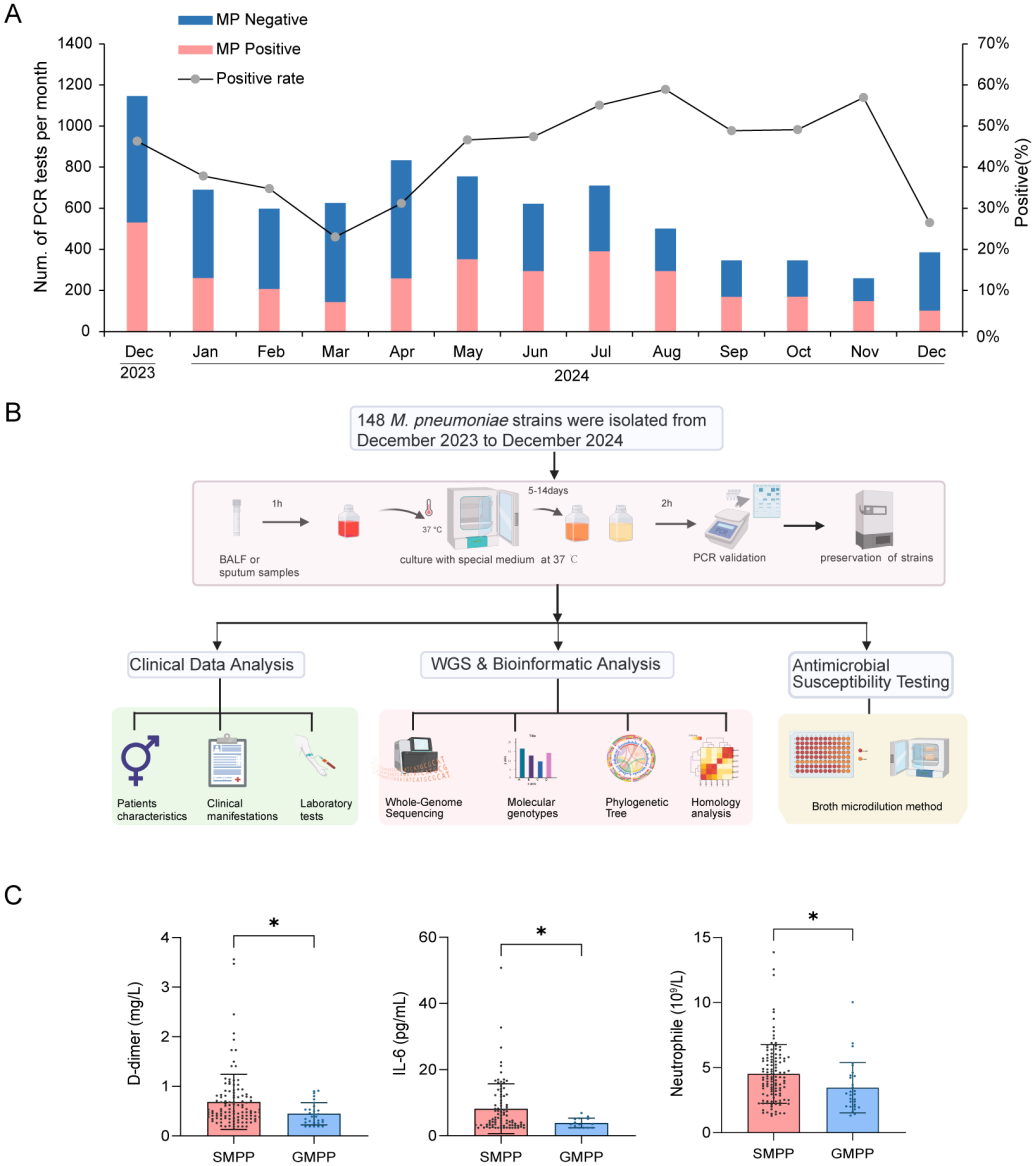
Epidemiological trends of *Mycoplasma pneumoniae* strains from pediatric patients with suspected respiratory infections. **(A)** Monthly average positive rate and number of PCR testing for suspected *M. pneumoniae* infections at Shanghai Children’s Hospital (2023.12–2024.12). **(B)** Study workflow. **(C)** Differences in key laboratory indicators between SMPP and GMPP group. *P < 0.05 (Student’s *t* test).

Our study indicated that the positive rate of *M. pneumoniae* gradually decreased from post-December 2023 to April 2024. After that, it increased until November 2024, stabilizing around 50%, and then declined again in December (**Figure 1A**). The infections predominantly occurred during the summer and autumn seasons, peaking in November 2024. To investigate the genomic characteristics of *M. pneumoniae* during the same period, we included 148 patients with *M. pneumoniae* infections (**Figure 1B**), mainly children aged 6–10 years (56.1%, 83/148), with a higher incidence observed among females (59.46%, 88/148). The isolates were mainly obtained from bronchoalveolar lavage fluid (93.24%, 138/148), and the others were derived from sputum samples. The number of collected strains also peaked in the summer and autumn of 2024, and was significantly correlated with the PCR positive rate (**Supplementary Figure 1**).

All patients presented fever and cough. Based on clinical diagnosis by pediatricians, the *M. pneumoniae* isolates were classified into SMPP (79.7%, 118/148) and general *M. pneumoniae* pneumonia (GMPP) (20.4%, 30/148) (**Table 1**). Statistical analysis revealed no significant differences between the two groups in term of age, gender, hospital duration, post-admission fever, wet rales, maximum temperature, respiratory rate, extent of pulmonary involvement and incidence of co-infection (**Table 1**). However, children diagnosed with SMPP demonstrated a higher incidence of extrapulmonary manifestations (23.7% vs 6.7%, *P* < 0.05) and were more likely to receive minocycline therapy (93.2% vs 66.7%, *P* < 0.001; **Table 1**). Laboratory findings indicated that SMPP patients had higher levels of absolute neutrophil counts, D-dimer, and interleukin-6 in peripheral blood (*P* < 0.05, **Figure 1C**, **Supplementary Table 1**).

**Table 1:** Demographic and clinical characteristics of children with severe *Mycoplasma pneumoniae* pneumonia and general *Mycoplasma pneumoniae* pneumonia, Shanghai, 2023-2024*.

Characteristics		SMPPƗ (n=118)	GMPPƗ (n=30)	P valueǂ
Demographic	Age	6.75 ± 2.74	6.17 ± 2.57	0.27
	<3	7(5.93%)	1(3.33%)	–
	3-5	30(25.42%)	13(43.33%)	–
	6-10	69(58.47%)	14(46.67%)	–
	>10	12(10.17%)	2(6.67%)	–
	Gender(male)	47(39.83%)	12(40%)	0.99
Clinical features	Fever post-admission	2.03 ± 1.77	1.56 ± 1.50	0.20
	Hospital duration	6.20 ± 2.11	5.97 ± 2.20	0.60
	Wet rale	41(34.75%)	11(36.67%)	0.32
	Max temp	38.43 ± 1.06	38.24 ± 0.99	0.40
	Respiratory rate	25.47 ± 3.29	24.81 ± 3.32	0.35
	Unilateral Lobar	88(74.58%)	20(66.67%)	0.49
	Bilateral Lobar	29(24.58%)	8(26.67%)	0.82
Intrapulmonary manifestations	Total	59(50%)	12(40%)	0.88
	Pleural effusion	33	7	0.61
	Plastic bronchitis	11	1	–
	Consolidation	12	1	–
	Bronchiectasis	3	3	–
Extrapulmonary manifestations	Total	28(23.73%)	2(6.67%)	0.03
	Gastrointestinal tract involvement	10		–
	Dermatitis	4		–
	Hepatic dysfunction	4	1	–
	Cardiac involvement	2	1	–
	Nephritis	1		–
	Coagulopathy	7		–
Co-infection	Virus and/or bacteria	42(35.59%)	12(40%)	0.65
Treatment	Minocycline	110(93.22%)	20(66.67%)	<0.001

^*^ Continuous variables were expressed as means ± standard deviation. Categorical variables were expressed as percentages.

^Ɨ^ SMPP, severe *Mycoplasma pneumoniae* pneumonia; GMPP, general *Mycoplasma pneumoniae* pneumonia.

^ǂ^ Continuous variables were compared using Student *t* test, and categorical variables were compared using Chi-square test or Fisher’s exact test.

### 3.2 Molecular types

At first, we conducted whole-genome sequencing and molecular typing on all 148 *M. pneumoniae* isolates. These isolates were classified into two P1 genotypes, eleven MLVA types and two sequence types (STs). The P1–1 genotype was predominant, accounting for 92.57% of isolates (n=137). MLST analysis identified ST3 as the most common sequence type (88.51%, n=131), followed by ST14 (6.76%, n=10). MLVA typing indicated that the M4-5-7–2 genotype (79.73%, n=118) was most prevalent, with M3-5-7–2 being the second most common (5.41%, n=8) (**Table 2**).

**Table 2:** Summary of different molecular types of 148 *Mycoplasma pneumoniae* isolates.

Genotypes		Case No. (n)	Percentage (%)
P1	P1-1	137	92.57
	P1-2	11	7.43
MLST	ST3	131	88.51
	ST14	10	6.76
	Undefined	7	4.73
MLVA	M4-5-7-2	118	79.73
	M3-5-7-2	8	5.41
	M4-3-7-2	4	2.70
	M4-4-7-2	4	2.70
	M3-5-6-2	4	2.70
	M3-5-4-2	3	2.03
	M3-5-1-2	2	1.35
	M4-5-5-2	2	1.35
	M4-2-7-2	1	0.68
	M3-5-5-2	1	0.68
	M3-2-4-2	1	0.68

**Figure 2:**
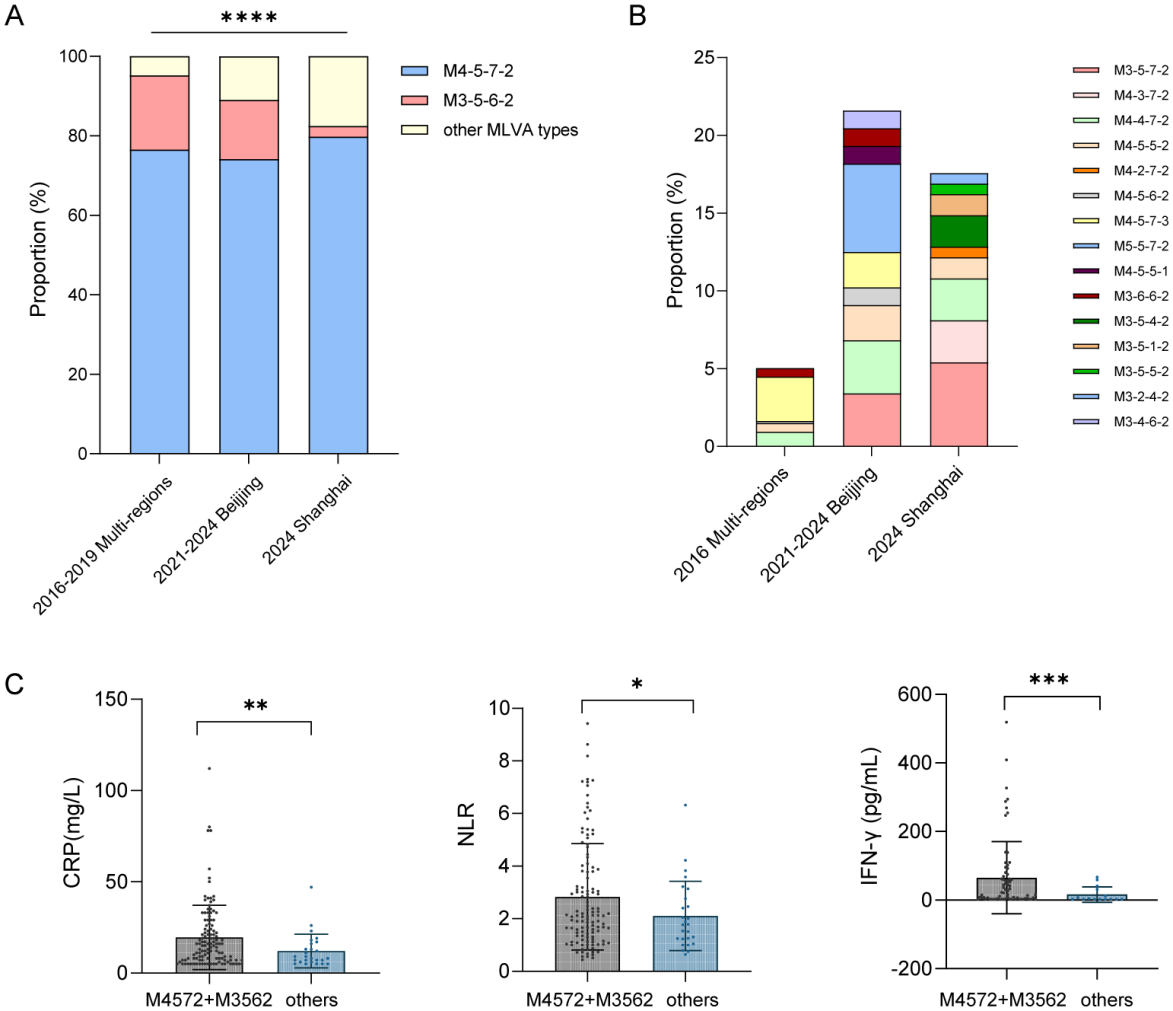
Shifting profiles of *Mycoplasma pneumoniae* MLVA types in China (2016–2024). **(A)** Distribution of major MLVA types across multiple Chinese cities before and after the COVID-19 pandemic (2016–2024). The “Other MLVA types” category excludes M4-5-7–2 and M3-5-6-2. Data for 2016–2019 were compiled from multi-regional studies (references Jiao et al., 2025; Wang et al., 2022; Xue et al., 2018; Zhao et al., 2019). Data for 2021–2023 in Beijing were obtained from references Jiao et al., 2025; Chen et al., 2024; Jia et al., 2024. The 2024 Shanghai data are from the present study. ****, *P* < 0.0001 (Fisher’s exact test). **(B)** Detailed proportional composition of the “Other MLVA types” category shown in panel **(A, C)** Differences in laboratory indicators between the common MLVA types (M4-5-7–2 and M3-5-6-2) and other MLVA types. *, *P* < 0.05. **, *P* < 0.01. ***, *P* < 0.001 (Student’s *t* test).

To better understand the recent trends in genotype changes of *M. pneumoniae*, we performed an integrated comparison of the published MLVA types from before and after the COVID-19 pandemic (**Supplementary Table 2**) (Jiao et al., 2025; Wang et al., 2022; Xue et al., 2018; Zhao et al., 2019; Chen et al., 2024; Jia et al., 2024). Our findings revealed that the prevalence of the M3-5-6–2 type accounted for 18.69% (137/733) of cases in multiple Chinese cities during 2016-2019, while the uncommon MLVA genotypes constituted only 4.77% (35/733). In contrast, we found a notable decrease in the prevalence of the M3-5-6–2 type, while the proportion of uncommon MLVA types, such as M3-5-7–2 and M4-4-7-2, increased markedly after the COVID-19 pandemic, reaching 17.57% (26/148) in our study (*P* < 0.0001, **Figure 2A**, **Table 2**). Moreover, these rare MLVA types exhibited substantial genotypic diversity (**Figure 2B**). To investigate whether this shift in MLVA types is associated with distinct clinical features, we compared the clinical manifestations, laboratory indicators, and drug sensitivity results between patients with the common types (M4-5-7–2 and M3-5-6-2) and those with rare MLVA types. Our analysis revealed significantly lower levels of CRP, neutrophil-to-lymphocyte ratio (NLR), and interferon-gamma (IFN-γ) in peripheral blood among patients infected with rare MLVA types compared to those with common types (**Figure 2C**, **Supplementary Table 3**).

**Figure 3:**
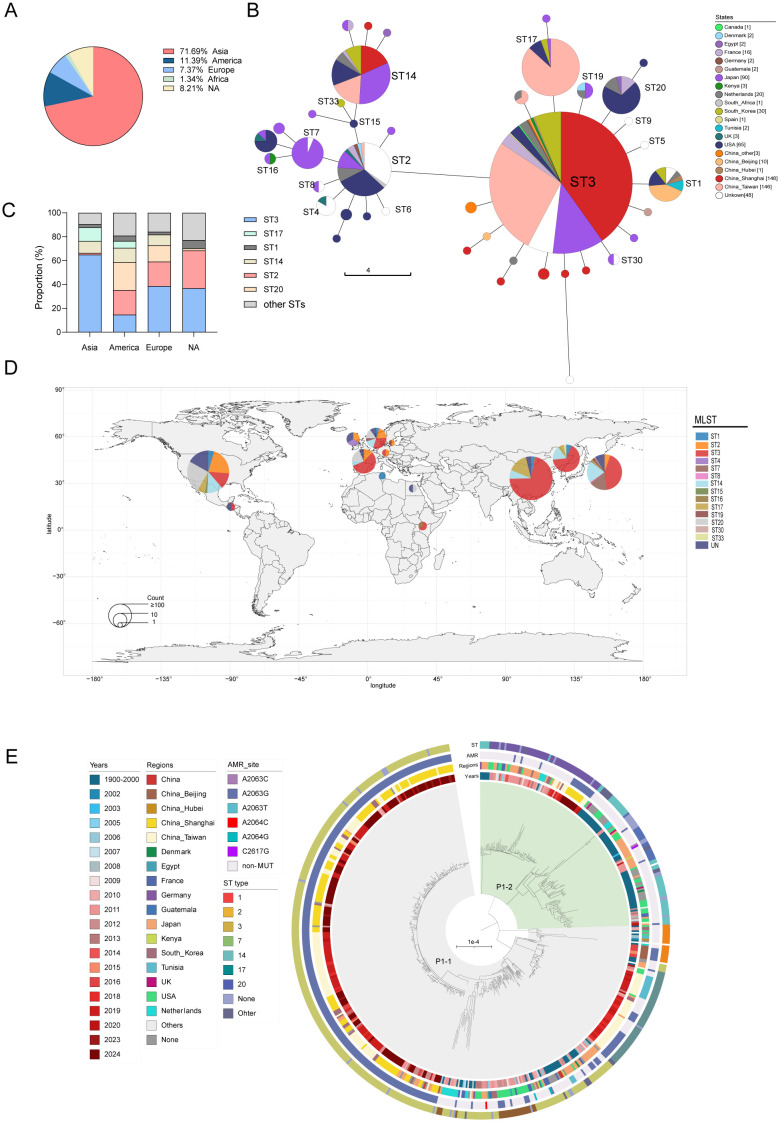
Population structure and geographical distribution of *Mycoplasma pneumoniae* sequence types. **(A)** Combined dataset of 597 *M. pneumoniae* genomes across different continents. NA, not assigned. **(B)** The minimum spanning tree of the 597 *M. pneumoniae* genomes based on STs. Each circle represents a unique ST type and the circle size is proportional to the number of isolates. Colors represent geographical regions. Samples from the current study are marked in red. **(C)** Prevalence of major STs across different continents. **(D)** Geographical distribution of ST types among 597 isolates (including 148 from this study). Pie charts depict the ST composition per region, with chart size proportional to the number of isolates. **(E)** Phylogenetic tree of the 148 *M. pneumoniae* isolates. Metadata for isolation year, geographical region, antimicrobial resistance (AMR) genes, and ST types were coded in the outside rings by colors.

Then, we performed a phylogenetic analysis of MLST profiles for 597 globally distributed *M. pneumoniae* isolates. The dataset was composed of 449 whole genome sequences from NCBI, sourced from Asia (n=280), America (n=68), Europe (n=44), Africa (n=8), and unknown regions (n=49), alongside 148 clinical isolates sequenced in this study (**Supplementary Dataset 1**). The majority of genomes originated from Asia (**Figure 3A**). The minimum spanning tree of STs were clustered into two major phylogenetic groups globally. One predominantly containing ST3, ST17, ST1, and ST20, and the other mainly consisting of ST14, ST2, and ST7 (**Figure 3B**). Notable geographic variations were observed in STs distribution, with ST3 (P1-1), ST17 (P1-1), and ST14 (P1-2) primarily disseminating in Asia, while ST2 (P1-2), ST20 (P1-1) and ST3 (P1-1) predominantly spreading in Europe and the Americas (**Figures 3C, D**, **Supplementary Figure 2**). Interestingly, ST20, classified as P1–1 type, was predominantly observed in western regions.

The phylogenetic tree showed all of genomes were broadly classified into two principal clades, P1–1 and P1-2 (**Figure 3E**). Most of our isolates clustered within the P1–1 clade and demonstrated closer phylogenetic relationships with strains from other Chinese cities, followed by isolates from Japan, South Korea, and the Netherlands.

### 3.3 Phenotypic and genotypic results of macrolides resistance

Results from the broth microdilution assay demonstrated that all isolates exhibited high-level resistance to erythromycin and clindamycin (Minimum Inhibitory Concentration, MIC range: 64->128 mg/L), followed by azithromycin (MIC range: 2–64 mg/L), and relatively lower resistance to 16-membered macrolides, midecamycin and josamycin (MIC range: 0.5–8 mg/L). In contrast, tetracyclines and fluoroquinolones showed excellent *in vitro* activity (MICs ≤ 0.5 mg/L), with minocycline, doxycycline, and moxifloxacin presenting the lowest MIC_50_ values (0.064 mg/L) (**Table 3**). Detailed MIC distribution and cumulative percentage was provided in **Supplementary Table 4** and **Supplementary Figure 3A**. Although all strains harbored the A2063G macrolide-resistant mutation, we found that P1–1 genotype isolates exhibited significantly higher MIC values for azithromycin (*P* < 0.05; **Supplementary Table 5**) and 16-membered macrolides (midecamycin and josamycin) compared to P1–2 genotype isolates (*P* < 0.001; **Supplementary Table 5**; **Supplementary Figure 3B**).

**Table 3:** Summary of the minimum inhibitory concentrations of ten antimicrobials against 146 *Mycoplasma pneumoniae* isolates*.

Antibiotics	Interpretive criteriaƗ		MIC (mg/L)				
	Susceptible	Resistant	Range	MIC50	MIC90	Susceptible%	Resistant%
Erythromycin	≤0.5	≥1	64->128	>128	>128	0	100
Clindamycin	–	–	64->128	128	>128	–	–
Azithromycin	≤0.5	≥1	2-64	16	32	0	100
Midecamycin	–	–	1-8	2	4	–	–
Josamycin	–	–	0.5-8	2	4	–	–
Tetracycline	≤2	–	0.06-0.5	0.25	0.25	100	0
Minocycline	–	–	<0.03-0.25	0.06	0.125	–	–
Doxycycline	≤2	–	<0.03-0.25	0.06	0.125	100	0
Levofloxacin	≤1	–	0.125-0.5	0.25	0.25	100	0
Moxifloxacin	≤0.5	–	<0.03-0.25	0.06	0.06	100	0

^*^ Antimicrobial susceptibility testing was successfully performed on 146 of 148 isolates. MIC, minimal inhibitory concentration.

^Ɨ^ Interpretive criteria from the CLSI M43-A.

To further compare the variation pattern of antibiotics resistance in *M. pneumoniae*, we reviewed previous studies on antibiotics resistance phenotypes, including 13 articles reporting MICs for macrolides, tetracyclines and fluoroquinolones (**Supplementary Table 6**). Recent comprehensive studies from multiple Chinese cities, as well as data from Beijing and Japan, indicate that the MIC_50_ of azithromycin ranged from 32 to 64 mg/L. In contrast, isolates in our study showed a slightly lower level of resistance to this antibiotic with the MIC_50_ of 16 mg/L. Based on our research data and published data, the MIC_50_ of tetracycline ranged from 0.25 to 0.5 mg/L, MIC_50_ of minocycline ranged from 0.06 to 0.5 mg/L, and MIC_50_ of levofloxacin ranged from 0.25 to 1 mg/L. To date, MICs of all tested strains for both tetracyclines and fluoroquinolones were below the resistance breakpoint criteria.

Analysis of macrolides-resistance mutation revealed that the A2063G mutation of 23S rRNA gene was presented in all of isolates. Only one strain additionally carried the C385T (V129I) mutation in the L4 ribosomal protein gene, yet this did not result in an increase of MICs. From a global perspective, *M. pneumoniae* with macrolide resistance mutations mainly disseminated in Asian, and the A2063G mutation of 23S rRNA gene was the most prevalent (**Supplementary Figure 3C**).

### 3.4 Virulence analysis and homology analysis

**Figure 4:**
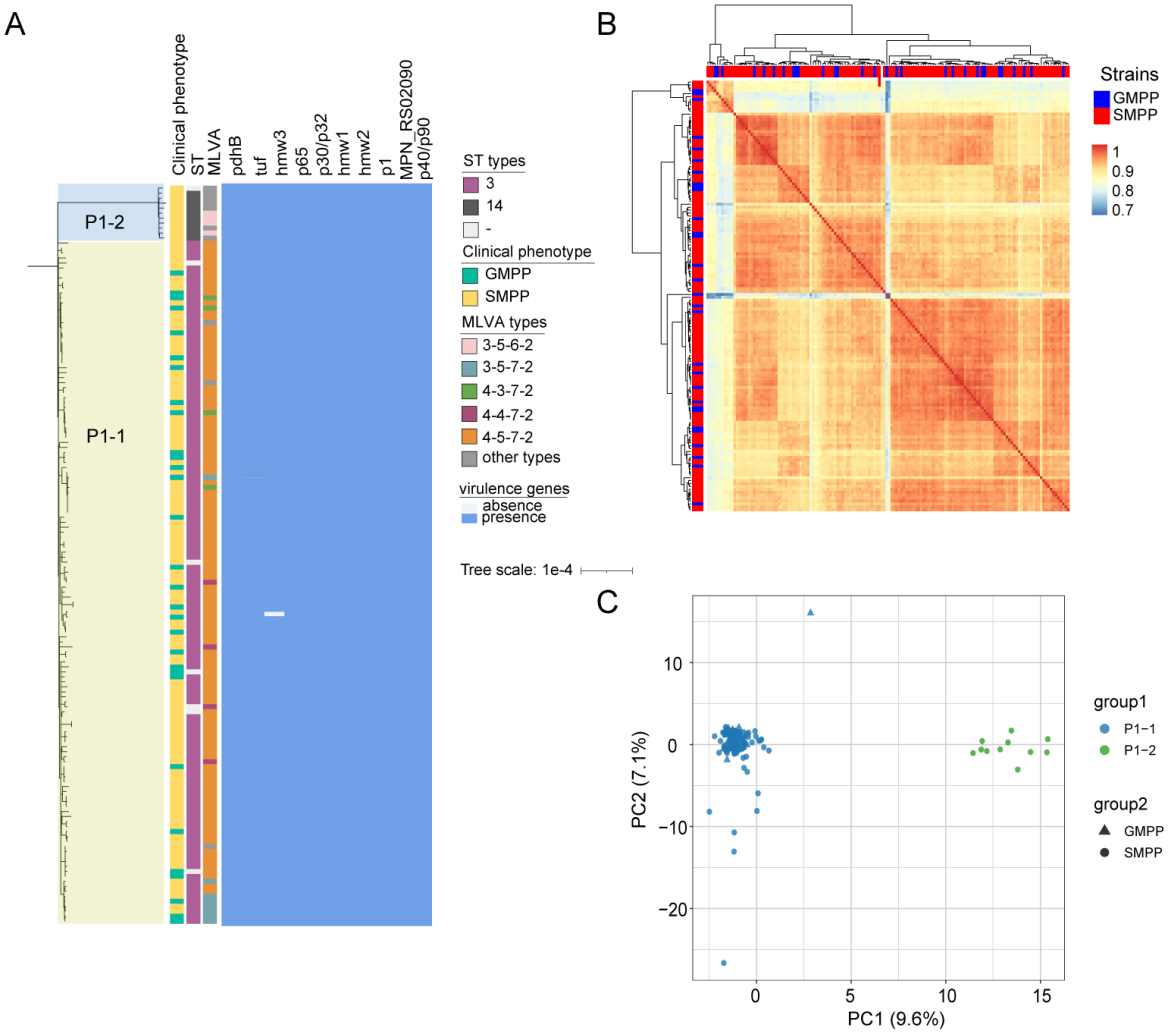
Comparative genomics analysis of SMPP and GMPP-associated *Mycoplasma pneumoniae* strains. **(A)** Phylogenetic tree (left) and virulence gene profile (right) of the 148 *M. pneumoniae* isolates. **(B)** Pairwise nucleotide divergence heatmap of all isolates. **(C)** PCA based on genome-wild SNP profiles. Isolates were visualized by P1 genotypes (color) and disease severity (shape).

Ten virulence genes were detected, including attachment organelle component (*P1*, *hmw1, hmw2, hmw3, P65, P30/P32*, *P40/P90*), *tuf* (elongation factor Tu), *phdB* (pyruvate dehydrogenase subunit beta), and *MPN_RS02090* (encoding community-acquired respiratory distress syndrome). All isolates contained all ten virulence genes (**Figure 4A**). The heatmap of the correlation analysis of *M. pneumoniae* genomes showed high similarity across different strains, while notably, the GMPP and SMPP strains failed to cluster separately (**Figure 4B**). However, PCA of whole-genome sequences clearly distinguished these strains according to P1 genotype (**Figure 4C**).

**Figure 5:**
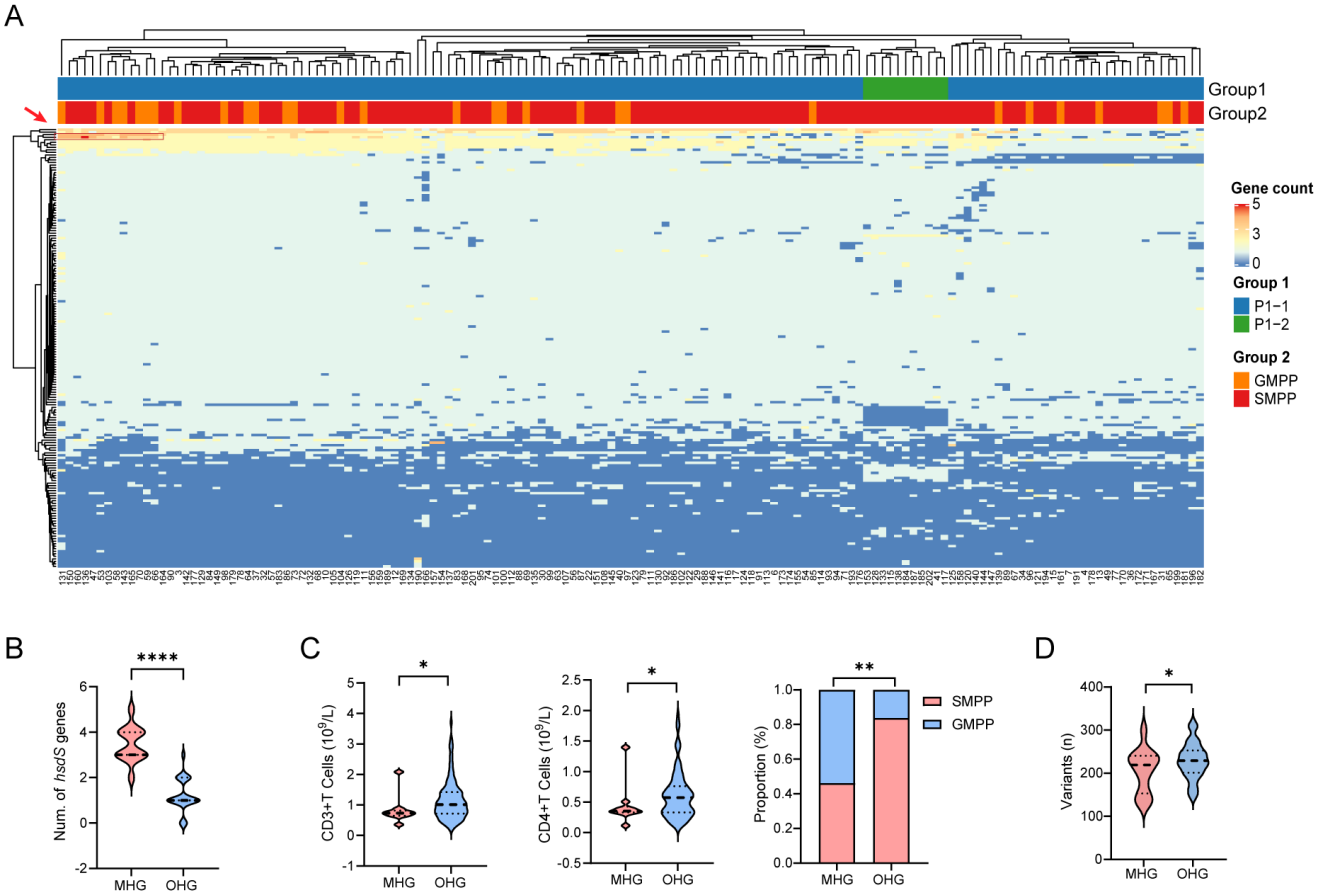
Genotypic and phenotypic comparison of *hsdS* homologs. **(A)** Heatmap showing homologous gene clusters across *M. pneumoniae* strains. Columns represent strains and rows represent homologous gene clusters. Group 1 denotes the P1 classification, while Group 2 indicates the disease severity. The red arrow and box highlight the MHG group with more *hsdS* homologous genes. **(B)** The number of *hsdS* homologs per isolate from the MHG and OHG groups. **(C)** Clinical features of pediatric patients infected by *M. pneumoniae*. **(D)** Burden of genomic variation in isolates from the MHG and OHG groups. Fisher’s exact test or Mann-Whitney *U* test was used as appropriate., *, *P* < 0.05. **, *P* < 0.01. ****, *P* < 0.0001.

Homologous genes typically perform similar functions. To investigate whether the amplification of homologous genes at the genomic level is related to the clinical manifestations of *M. pneumonia* infection, we conducted an orthologous gene family analysis across 148 genomes. A total of 1,133 homologous gene clusters were identified. In the heatmap of homologous clusters (**Figure 5A**), we observed that within the 13 strains on the left, there was a particular homologous cluster that containing a significantly larger number of homologous genes. We classify samples with a high number of homologous genes as multi-homologous gene groups (MHG), and other samples as oligo-homologous gene groups (OHG) (**Figure 5B**). Further analysis revealed that specific gene MPN365 and MPN615 in this homologous cluster exhibited a significantly higher occurrence rate in the MHG (**Table 4**). Through BLAST alignment, we found that these genes belong to a family of restriction endonuclease subunit S (*hsdS*). Previous studies have demonstrated that the tandem repeats within certain MPN gene, such as *hsdS*, were correlated with macrolide resistance (Lee et al., 2022). However, no significant difference in the frequency of the tandem repeat sequence CCGAGCTAAGCG within each MPN gene was observed between MHG and OHG (**Table 4**). Clinical data analysis showed that patients infected with strains from MHG had lower peripheral blood CD3+ and CD4+ lymphocytes counts and were more frequently diagnosed with GMPP (**Figure 5C**, **Supplementary Table 7**) (*P* < 0.01). Moreover, these strains exhibited a significantly lower burden of genomic variations compared to others strains (*P* < 0.05, **Figure 5D**). In addition, genotype-specific differences were also found in the paralog counts of two genes: the Holliday junction helicase (*RuvB*) and DNA polymerase III subunit C (*polC*) (**Supplementary Dataset 1**). These genes were implicated in homologous recombination and genomic replication fidelity, respectively. The expansion patterns were genotype-specific: P1–1 isolates possessed 3 *RuvB* and 6 *polC* paralogs, whereas P1–2 strains carried 2 *RuvB* and 7 *polC* paralogs (**Table 5**).

**Table 4:** Proportion of samples containing *hsdS* genes per group and average tandem repeat numbers of each *hsdS* gene between multi-homologous gene groups and oligo-homologous gene groups*.

MPN genes	MHG (n=13)		OHG (n=135)		TR numbers in M129 strain^Ɨ^
	% *hsdS* gene containing samples	Average TR numbers	% *hsdS* gene containing samples	Average TR numbers	
MPN201	NDǂ	ND	11.1	0	0
MPN258	7.7	16	3.0	14.5	15
MPN289	92.3	3.2	74.1	4.4	4
MPN343	38.5	9.6	14.8	9.4	16
MPN365	100	0	4.4	0	1
MPN615	100	4.7	14.8	1.95	1

*Multi-homologous gene groups (MHG) and oligo-homologous gene groups (OHG); tandem repeat (TR).

^Ɨ^ Tandem repeat copy numbers of each MPN in M129 strain resourced from Li et al.’s report (Xiao et al., 2015).

^ǂ^ ND, not detected.

**Table 5:** Paralog counts of the gene of *RuvB* and *polC* in P1–1 and P1–2 isolates.

Genes	Paralog counts		Gene start*	Gene end*	Annotation	UniProtKB
	P1-1	P1-2				
*RuvB*	3	2	162240	163163	Holliday junction branch migration, DNA helicase	A0A0H3DN66
*polC*	6	7	774405	778736	DNA polymerase III subunit alpha	A0A0H3DK82

*NZ_LR214945.1 Reference assembly.

## 4 Discussion

In this study, we isolated 148 *M. pneumoniae* strains at Shanghai Children’s Hospital from December 2023 to December 2024, providing a comprehensive analysis of clinical features, MICs of ten antibiotics, molecular epidemiology and genomic characteristics of clinical isolated *M. pneumoniae*.

Previously studies had reported that SMPP accounted for approximately 12% of all MPP cases (Gao et al., 2019; Zhang et al., 2024). However, recent post-COVID-19 research had shown a variable but notable increase in SMPP proportions among children, ranging from 20% (Zhang et al., 2024; Jiang et al., 2023) to 40-50% (Qiu et al., 2024; Yang et al., 2024; Chen et al., 2024), and even exceeding 70% (Jiao et al., 2025). In our study, 79.7% (118/148) of patients were diagnosed as SMPP. We speculated several factors may contribute to this high SMPP prevalence. Frist, reduced exposure to *M. pneumoniae* during COVID-19 pandemic may have led to diminished population immunity, particularly among children, which was called as “immunity debt” (Zhang et al., 2024). Second, all strain, regardless of genotype, exhibited macrolide resistance, which may exacerbate disease severity. Third, the majority of our strains were M4-5-7-2, which has been reported as carrying more virulence-related genes and enriched genes supporting cellular processes and metabolism (Jiao et al., 2025).

To provide clinicians with information on the pathogen’s sensitivity to commonly used antibiotics in the post-COVID-19 era, we conducted susceptibility tests. Compared to data collected five years ago (Wang et al., 2022), MICs of erythromycin and clindamycin remained persistently high and the MIC_50_ of azithromycin remained unchanged, while the MIC_50_ for midecamycin, josamycin, tetracyclines, and fluoroquinolones has decreased by 1–2 dilution steps. In China, the extremely high prevalence of MRMP in the P1–1 genotype has become widely recognized. Prior to the COVID-19 pandemic, the P1–2 genotype exhibited a relatively low rate of macrolide resistance (Wang et al., 2022). However, multiple studies conducted before and after the pandemic—based on detection of 23S rRNA mutations—have reported a progressive increase in the macrolide resistance mutation rate of the P1–2 type (Wang et al., 2022; Jiang et al., 2023; Chen et al., 2024; Wang et al., 2021, 2022). This rate has now surged to an alarming 90%-100%. In the present study, although both P1–1 and P1–2 strains carried the A2063G resistance mutation at 100% prevalence, the P1–2 genotype strains exhibited significantly lower MICs to azithromycin and 16-membered macrolides than P1–1 genotype strains. The divergence of molecular and phenotypic resistance highlights the importance of *in vitro* antimicrobial susceptibility testing in providing valuable guidance for clinical antibiotic use. Nevertheless, given that the rate of MRMP in Shanghai, China, has now reached nearly 100%, there is an urgent need for enhanced antibiotic management strategies and evidence-based clinical guidelines to strengthen antimicrobial stewardship and mitigate the growing threat of macrolide resistance.

In the present study, the proportion of uncommon MLVA genotypes surpassed that of M3-5-6-2. To our knowledge, this study was the first to report an increase in less common *M. pneumoniae* genotypes after COVID-19 pandemic. We speculated that during the COVID-19 pandemic, the overall antibody level in children across all age groups against *M. pneumoniae* significantly declined (Liu et al., 2024), resulting in reduced selective pressure on *M. pneumoniae*, which in turn facilitated their rapid dissemination and led to increased *M. pneumoniae* diversity during the subsequent resurgence. Although we did not observe a different MRMP prevalence or clinical manifestations in uncommon subtypes, we found that patients infected with these strains exhibited lower levels of CRP, NLR and IFN-γ compared to those infected with M4-5-7–2 and M3-5-6-2. Nevertheless, the proportion of SMPP due to the uncommon types remained as high as 73%, suggesting distinct immune mechanisms evoked by these strains. In summary, the population structure of *M. pneumoniae* has undergone significant changes possibly associated with the COVID-19 pandemic, highlighting ongoing monitoring of its transmission dynamics.

Previous research had demonstrated that *hsdS* gene participates in restriction-modification (R-M) systems, which were widely distributed across diverse bacterial species and played a crucial role in regulating DNA epigenetic modification (De Ste Croix et al., 2017). These DNA methylation events modulated bacterial pathogenicity by influencing the expression of transcription factors (Ma et al., 2025). In *Mycoplasma hominis*, methylation mediated by R-M systems had been shown to be involved in regulating host adaptation mechanisms (Pobeguts et al., 2024). The tandem repeats in *hsdS* may be associated with macrolide resistance, meaning that *hsdS* itself influences the phenotypes of *M. pneumoniae* (Lee et al., 2022). In this study we observed that strains with a higher abundance of *hsdS* genes caused less severe symptoms and were associated with lower levels of peripheral CD3+ and CD4+ T lymphocytes. Therefore, we supposed that epigenetic regulation in these strains modulates the expression of virulence genes, enabling the *M. pneumoniae* to adapt to the host environment in a less virulent state. In line with this, the frequency of whole genome mutations of strains in MHG was also relatively lower. Nevertheless, further functional characterization of these genes need be pursued in future studies.

In conclusion, we isolated and characterized pediatric *M. pneumoniae* strains in Shanghai between late 2023 and 2024. Whole-genome sequence and antimicrobial susceptibility testing revealed a shifting landscape of MLVA genotypes and highlighted the continued high prevalence of the A2063G mutation across all strains. Notably, the P1–2 strains demonstrated a lower level of macrolides resistance than P1–1 strains. In addition, the proportion of clinical severe pneumonia cases was higher than ever before, a trend likely influenced by widespread macrolide resistance and the COVID19−pandemic. Finally, the significant genomic divergence between strains, represented by *hsdS* genes homologs, which is associated with the severity of *M. pneumoniae* pneumonia, reflected ongoing bacterial evolution in the context of pathogen-host interactions.

## Data Availability

The datasets presented in this study can be found in online repositories. The names of the repository/repositories and accession number(s) can be found in the article/**Supplementary Material**. All whole-genome sequencing data from the study are available through the Sequence Read Archive in National Centre for Biotechnology Information via the project accession number RJNA1286769.
