# Supplementary material for: Genomic epidemiology and antimicrobial susceptibility of clinical Mycoplasma pneumoniae isolates in Shanghai, 2023–2024
Source: Front Cell Infect Microbiol. 2026 Jul 1;16:1747629. doi: 10.3389/fcimb.2026.1747629 (PMC13368468; doi:10.3389/fcimb.2026.1747629)
Supplement: Supplementary file 1 [file Table1.docx]

Supplementary Material

# Supplementary Figures and Tables

## Supplementary Figures

**Supplementary Figure 1.** Sample collection for 148 strains. (A) Number of strains collected per month. (B) Correlation between number of strains collected per month and PCR positivity rate of *Mycoplasma pneumoniae.* PCR positivity rate data were derived from Figure 1A.

**Supplementary Figure 2.** Temporal changes in sequence types of *Mycoplasma pneumoniae* across regions and time period (1940-2024).

**Supplementary Figure 3.** Drug-resistance features of *M. pneumoniae*. (A) Cumulative bacteriostatic rates (%) of the ten antimicrobials against 146 *M. pneumoniae* isolates. Colors represent different antibiotics. The dashed lines denote MIC₅₀ (lower) and MIC₉₀ (upper), respectively. (B) Differences in resistance to 16-membered macrolides between P1-1 and P1-2 genotypes. Mann-Whitney *U* test. ****, *P* < 0.0001. (C) Global distribution of *M. pneumoniae* 23S rRNA mutations. Pie charts show 23S rRNA mutations of 597 *M. pneumoniae* isolates (148 from this study) from various countries/regions. The chart size represents the number of isolates.

## Supplementary Tables

Table S1. Laboratory indicators in peripheral blood of children with severe *Mycoplasma pneumoniae* pneumonia and general *Mycoplasma pneumoniae* pneumonia, Shanghai, 2023-2024*

| Laboratory indicators | SMPPƗ (n=118) | GMPPƗ (n=30 | *P* value ǂ |
| --- | --- | --- | --- |
| WBC (10^9^/L) | 7.14±3.01 | 6.12±2.31 | 0.086 |
| Neutrophile (10^9^/L) | 4.53±2.27 | 3.43±1.91 | 0.048 |
| Lymphocyte (10^9^/L) | 2.02±1.29 | 2.09±1.08 | 0.80 |
| NLR | 3.26±5.05 | 2.19±1.92 | 0.26 |
| CRP (mg/L) | 19.36±17.96 | 14.03±9.29 | 0.12 |
| ESR (mm/L) | 34.48±19.71 | 36.37±23.25 | 0.65 |
| MPV (fL) | 9.54±0.84 | 11.93±13.58 | 0.55 |
| D-D (mg/L) | 0.73±0.74 | 0.45±0.23 | <0.001 |
| FG (g/L) | 4.10±0.54 | 3.9±0.53 | 0.082 |
| SF (ng/mL) | 127.66±183.42 | 84.26±45.36 | 0.26 |
| LDH (U/L) | 362.3±100.03 | 329.77±87.93 | 0.11 |
| CD3 (10^9^/L) | 1.10±0.57 | 1.30±0.95 | 0.37 |
| CD4 (10^9^/L) | 0.59±0.37 | 0.65±0.44 | 0.52 |
| CD8 (10^9^/L) | 0.42±0.22 | 0.51±0.44 | 0.34 |
| IL-1β (pg/mL) | 10.67±9.32 | 7.86±4.76 | 0.26 |
| IL-6 (pg/mL) | 8.23 ±7.59 | 3.85±1.45 | <0.001 |
| IL-8 (pg/mL) | 15.56±23.7 | 6.84±7.71 | 0.16 |
| IL-10 (pg/mL) | 2.70±1.12 | 2.54±0.27 | 0.59 |
| IL-17 (pg/mL) | 4.81±3.03 | 6.00±4.97 | 0.38 |
| TNF-α (pg/mL) | 3.24±3.51 | 3.14±1.11 | 0.92 |

*Continuous variables were expressed as mean ± standard deviation. WBC, white blood cell; NLR, neutrophil-to-lymphocyte ratio; CRP, neutrophil-to-lymphocyte ratio; ESR, erythrocyte sedimentation rate; MPV, mean platelet volume; D-D, D-dimer; FG, fibrinogen; SF, serum ferritin; LDH, lactate dehydrogenase.

ƗSMPP, severe *Mycoplasma pneumoniae* pneumonia; GMPP, general *Mycoplasma pneumoniae* pneumonia.

ǂ Continuous variables were compared using Student *t* test

Table S2. Prevalence of multi-locus variable number types in China before and after COVID-19 pandemic (Detailed data sources presented in Figure 2A)

|  | Before COVID-19 pandemic | | | | | After COVID-19 pandemic | | | |
| --- | --- | --- | --- | --- | --- | --- | --- | --- | --- |
|  | 2016 Six regions in China | 2017-2018 Five regions in China | 2017-2019 Shanghai | 2018-2019 Beijing | 2021-2023 Beijing | | 2023 Beijing | 2023-2024 Beijing | 2024 Shanghai |
| M4-5-7-2 | 248 (72.1%) | 107 (69.5%) | 167 (90.8%) | 39 (76.5%) | 38 (61.3%) | | 73 (73.7%) | 58 (86.6%) | 118 (79.7%) |
| M3-5-6-2 | 76 (22.1%) | 35 (22.7%) | 14 (7.6%) | 12 (23.5%) | 14 (22.6%) | | 17 (17.2%) | 4 (6.0%) | 4 (2.7%) |
| Other types | 20 (5.8%) | 12 (7.8%) | 3 (1.6%) | 0 (0%) | 10 (16.1%) | | 9 (9.1%) | 4 (6.0%) | 26 (17.6%) |

Table S3. Demographic, clinical characteristics and laboratory indicators of children infected with rare and common MLVA types of *Mycoplasma pneumoniae*.*

| Characteristics | | Rare MLVA group (n=26) | Common MLVA group (n=122) | *P* valueǂ |
| --- | --- | --- | --- | --- |
| Demographic | Age | 6.48±3.40 | 6.66±2.54 | 0.76 |
|  | Gender(male) | 13(50.00%) | 47(38.52%) | 0.28 |
| Clinical features | SMPP | 19(73.08%) | 99(81.15%) | 0.35 |
|  | Fever post-admission | 1.81±1.60 | 1.96±1.76 | 0.67 |
|  | Hospital duration | 5.65±1.36 | 6.26±2.23 | 0.18 |
|  | Wet rale | 10(38.46%) | 42(34.45%) | 0.70 |
|  | Max temp | 38.24±1.04 | 38.42±1.05 | 0.43 |
|  | Respiratory rate | 24.65±2.31 | 25.50±3.45 | 0.24 |
| Pulmonary manifestations | Consolidation | 18(69.23%) | 89 (72.95%) | 0.094 |
|  | Extrapulmonary manifestations | 2(7.69%) | 25 (20.49%) | 0.125 |
| Co-infection | Virus and/or bacteria | 42(35.59%) | 12(40%) | 0.65 |
| Laboratory indicators | WBC (10^9^/L) | 6.36±2.40 | 7.04±2.99 | 0.27 |
|  | Neutrophile (10^9^/L) | 3.72±1.75 | 4.42±2.31 | 0.15 |
|  | Lymphocyte (10^9^/L) | 2.03±0.83 | 2.05±1.31 | 0.94 |
|  | NLR | 2.11±1.32 | 2.83±2.02 | 0.026 |
|  | CRP (mg/L) | 12.08±9.25 | 19.61±17.61 | 0.003 |
|  | ESR (mm/L) | 35.77±22.92 | 34.67±19.85 | 0.80 |
|  | MPV (fL) | 9.45±0.98 | 10.16±6.79 | 0.60 |
|  | D-D (mg/L) | 0.65±0.40 | 0.68±0.72 | 0.86 |
|  | FG (g/L) | 4.06±0.52 | 4.05±0.55 | 0.92 |
|  | SF (ng/mL) | 113.34±67.17 | 105.77±66.00 | 0.63 |
|  | LDH (U/L) | 358.77±80.93 | 355.05±101.49 | 0.86 |
|  | CD3 (10^9^/L) | 1.14±0.57 | 1.14±0.66 | 1.00 |
|  | CD4 (10^9^/L) | 0.60±0.34 | 0.60±0.39 | 0.98 |
|  | CD8 (10^9^/L) | 0.45±0.25 | 0.43±0.27 | 0.86 |
|  | IL-1β (pg/mL) | 9.39±10.30 | 10.67±8.79 | 0.58 |
|  | IL-6 (pg/mL) | 6.96 ±5.34 | 7.69±7.52 | 0.70 |
|  | IL-8 (pg/mL) | 19.02±33.00 | 13.40±19.44 | 0.33 |
|  | IL-10 (pg/mL) | 2.80±1.21 | 2.65±1.00 | 0.58 |
|  | IL-17 (pg/mL) | 4.52±2.75 | 5.07±3.47 | 0.53 |
|  | TNF-α (pg/mL) | 3.67±3.98 | 3.13±3.12 | 0.53 |
|  | IFN-γ (pg/mL) | 16.11±22.40 | 65.27±104.91 | < 0.001 |

* Common MLVA types refer to M 3-5-6-2 and M 4-5-7-2, while rare MLVA types refer to all MLVA types other than these two.

ǂContinuous variables were compared using Student *t* test, and categorical variables were compared using Chi-square test or Fisher’s exact test.

Table S4. The minimum inhibitory values of ten antibiotics against 146 *Mycoplasma pneumoniae* Isolates*

| Antibiotics | MIC value (mg/L) | | | | | | | | | | | | | |
| --- | --- | --- | --- | --- | --- | --- | --- | --- | --- | --- | --- | --- | --- | --- |
|  | <0.032 | 0.064 | 0.125 | 0.25 | 0.5 | 1 | 2 | 4 | 8 | 16 | 32 | 64 | 128 | >128 |
| Erythromycin |  |  |  |  |  |  |  |  |  |  |  | 3.4 | 20.5 | 100 |
| Clindamycin |  |  |  |  |  |  |  |  |  |  |  | 8.2 | 59.6 | 100 |
| Azithromycin |  |  |  |  |  |  | 1.4 | 11 | 50.1 | 87.8 | 99.3 | 100 |  |  |
| Medermycin |  |  |  |  |  | 2.7 | 50 | 95.2 | 100 |  |  |  |  |  |
| Josamycin |  |  |  |  | 0.7 | 9.6 | 71.9 | 98.6 | 100 |  |  |  |  |  |
| Tetracycline |  | 3.4 | 42.4 | 96.5 | 100 |  |  |  |  |  |  |  |  |  |
| Minocycline | 11.6 | 64.5 | 98.6 | 100 |  |  |  |  |  |  |  |  |  |  |
| Doxycycline | 6.2 | 52.1 | 93.8 | 100 |  |  |  |  |  |  |  |  |  |  |
| Levofloxacin |  |  | 0.7 | 99.3 | 100 |  |  |  |  |  |  |  |  |  |
| Moxifloxacin | 17.1 | 100 |  |  |  |  |  |  |  |  |  |  |  |  |

*Antimicrobial susceptibility testing was successfully performed on 146 of 148 isolates. MIC, minimal inhibitory concentration.

Table S5. Comparison of minimal inhibitory concentration between P1-1 and P1-2

| Antibiotics | MIC range (mg/L) | | MIC_50_/MIC_90_ (mg/L) | | *P* value (Whitney *U* test) |
| --- | --- | --- | --- | --- | --- |
|  | P1-1 | P1-2 | P1-1 | P1-2 |  |
| Erythromycin | 64->128 | 64->128 | >128/>128 | >128/>128 | 0.84 |
| Azithromycin | 2-64 | 4-32 | 16/32 | 8/8 | 0.026 |
| Clindamycin | 64->128 | 64->128 | 128/>128 | 128/>128 | 0.78 |
| Midecamycin | 1-8 | 1-2 | 4/4 | 2/2 | <0.001 |
| Josamycin | 1-8 | 1-2 | 2/4 | 1/2 | <0.001 |
| Tetracycline | 0.064-0.5 | 0.064-0.25 | 0.25/0.25 | 0.125/0.25 | 0.42 |
| Minocycline | <0.03-0.25 | <0.03-0.125 | 0.064/0.125 | 0.064/0.125 | 0.91 |
| Doxycycline | <0.03-0.25 | <0.03-0.25 | 0.125/0.125 | 0.125/0.25 | 0.88 |
| Levofloxacin | 0.125-0.5 | 0.25 | 0.25/0.25 | 0.25/0.25 | 1.0 |
| Moxifloxacin | <0.03-0.064 | <0.03-0.064 | 0.064/0.064 | 0.064/0.064 | 0.92 |

*MIC, minimal inhibitory concentration

Table S6. Key studies on *in vitro* antimicrobial susceptibility of clinical *Mycoplasma pneumoniae* isolates*

| Author | Publication | Isolation | Regions | Isolate Num. of *M. pneumoniae* | Macrolides,  MIC (mg/L) | Tetracyclines,  MIC (mg/L) | Fluoroquinolones,  MIC (mg/L) | Notes |
| --- | --- | --- | --- | --- | --- | --- | --- | --- |
| Waites. et al. | Antimicrob Agents Chemother, 2017 | 1980-2014 | United States, Europe, China | 42Ɨ | ERY:>32;  AZM:2->32;  SOLI:0.25-0.5 | TCY:0.25-1;  DOX:0.12-0.25 | MFX:0.125-0.25 | MIC Range |
| Jiang. et al. | Microbiology Spectrum, 2024 | 2003-2019 | China (Beijing) | 13 | ERY:>64 | NA | NA | MIC Range |
| Liu. et al. | Antimicrob Agents Chemother, 2009 | 2005-2008 | China (Shanghai) | 53 | ERY:>128;  AZM:16;  CLR:128;  JOS:2 | TCY:0.06;  DOX:0.06;  MNO:0.06 | LVX:0.5; MFX:0.106;  CIP:1 | MIC_50_ |
| Miyashita. et al. | Antimicrob Agents Chemother, 2013 | 2008-2012 | Japan | 125 | ERY:>128;  CLR:>128;  AZM:64;  ROK:0.25;  CLI:128 | TCY:0.5;  MNO:1 | TFX:0.25;  LVX:0.5;  MFX:0.0625;  GRN:0.0313 | MIC_50_ |
| Jiang. et al. | Epidemiology & Infection, 2024 | 2013-2019 | China (Seven regions) | 45 | ERY:>64;  AZM:>32 | TCY:<0.5 | LVX:<1 | MIC Range |
| Guo. et al. | Bosn J Basic Med Sci, 2019 | 2014 | China (Beijing) | 60 | ERY:128->1024;  AZM:16-256;  JOS:2-16 | TCY:0.063-0.25; MNO:0.032-0.25 | LVX:0.25-1 | MIC Range |
| Zhao. et al. | Antimicrob Resist Infect Control, 2019 | 2014-2016 | China (Beijing) | 53Ɨ | ERY:>256;  AZM:2-64 | TCY:0.016-0.5 | LVX:0.125-1 | MIC Rang |
| Zhao. et al. | Antimicrob Resist Infect Control, 2019 | 2017-2018 | China (Five regions) | 118 | ERY:128->256;  AZM:2-32 | TCY:0.016-0.25 | LVX:0.125-1 | MIC Range |
| Wang. et al. | Journal of Antimicrobial Chemother, 2020 | 2017-2018 | China (Shanghai) | 110 | ERY:>128;  AZM:8;  JOS:4;  MID:4 | TCY:0.5; MNO:0.5; DOX:0.25 | LVX:1 | MIC_50_ |
| Wang. et al. | Infect Drug Resist, 2022 | 2017-2019 | China (Shanghai) | 182 | ERY:>128;  AZM:16;  RXT:128;  JOS:4 | TCY:0.5; MNO:0.5;  DOX:0.25 | LVX:0.5;  MFX:0.125 | MIC_50_ |
| Oishi. et al. | Microorganisms, 2022 | 2017-2020 | Japan | 46Ɨ | CLR:>128;  AZM:64 | MNO:0.5 | TFX:0.25 | MIC_50_ |
| Jia. et al. | Front Cell Infect Microbiol, 2024 | 2021-2023 | China (Beijing) | 62 | ERY:512;  AZM:32;  ACE:0.25;  CHL:2 | TCY:0.25 | LVX:0.5 | MIC_50_ |
| Yan. et al. | JAC Antimicrob Resist, 2025 | 2023 | China (Ten regions) | 190 | ERY:512;  AZM:64 | TCY:0.5;  DOX:0.25 | MFX:<0.125 | MIC_50_ |

*ERY, Erythromycin； AZM, Azithromycin； JOS, Josamycin； CLR, Clarithromycin; ROK, Rokitamycin; CLI, Clindamycin; SOLI, solithromycin; RXT, Roxithromicin; ASP, Acetylspiramycin; CHL, Chloramphenicol; TCY, Tetracycline; DOX, Doxycycline; MNO, Minocycline; MFX, Moxifloxacin; LVX, Levofloxacin; CIP, Ciprofloxacin; TFX, Tosufloxacin; GRN, Garenoxacin; LEFA, Lefamulin

ƗThe number of macrolide resistance *M. pneumoniae* (MRMP) in those studies, and the minimum inhibitory concentrations (MICs) following it were only for MRMP.

Table S7. Demographic, clinical characteristics and laboratory indicators of children infected with OHG and MHG *M. pneumoniae.*

| Characteristics | | | OHG (n=135) | MHG (n=13) | *P* valueǂ |
| --- | --- | --- | --- | --- | --- |
| Demographic | Age | 6.60±2.65 | 6.92±3.28 | 0.68 |  |
|  | Gender(male) | 54(40.00%) | 6(46.15%) | 0.67 |  |
| Clinical features | SMPP | 112(82.96%) | 6(46.15%) | 0.002 |  |
|  | Fever post-admission | 2.00±1.58 | 1.92±1.75 | 0.88 |  |
|  | Hospital duration | 5.54±1.39 | 6.22±2.17 | 0.27 |  |
|  | Wet rale | 45(33.33%) | 7 (53.85%) | 0.14 |  |
|  | Max temp | 38.48±0.99 | 38.38±1.06 | 0.75 |  |
|  | Respiratory rate | 25.85±4.12 | 25.30±3.20 | 0.57 |  |
| Pulmonary manifestations | Consolidation | 96(71.11%) | 12(92.31%) | 0.002 |  |
|  | Extrapulmonary manifestations | 25(18.52%) | 2(15.38%) | 0.78 |  |
| Co-infection | Virus and/or bacteria | 42(35.59%) | 12(40%) | 0.65 |  |
| Laboratory indicators | WBC (10^9^/L) | 5.55±2.05 | 7.05±2.94 | 0.075 |  |
|  | Neutrophile (10^9^/L) | 3.32±1.89 | 4.39±2.25 | 0.10 |  |
|  | Lymphocyte (10^9^/L) | 1.70±0.64 | 2.08±1.27 | 0.30 |  |
|  | NLR | 2.19±1.50 | 2.75±1.97 | 0.32 |  |
|  | CRP (mg/L) | 20.08±20.04 | 18.09±16.37 | 0.68 |  |
|  | ESR (mm/L) | 37.00±19.29 | 36.66±20.51 | 0.68 |  |
|  | MPV (fL) | 9.58±0.67 | 10.08±6.47 | 0.78 |  |
|  | D-D (mg/L) | 0.69±0.44 | 0.67±0.39 | 0.92 |  |
|  | FG (g/L) | 4.22±0.51 | 4.04±0.54 | 0.26 |  |
|  | SF (ng/mL) | 131.90±87.23 | 105.01±94.95 | 0.22 |  |
|  | LDH (U/L) | 366.46±129.28 | 354.67±87.93 | 0.68 |  |
|  | CD3 (10^9^/L) | 0.84±0.46 | 1.16±0.65 | 0.023 |  |
|  | CD4 (10^9^/L) | 0.45±0.35 | 0.61±0.38 | 0.038 |  |
|  | CD8 (10^9^/L) | 0.31±0.11 | 0.44±0.27 | 0.055 |  |
|  | IL-1β (pg/mL) | 8.11±2.30 | 10.60±9.26 | 0.51 |  |
|  | IL-6 (pg/mL) | 7.58 ±9.40 | 7.57±7.09 | 0.99 |  |
|  | IL-8 (pg/mL) | 10.44±6.48 | 14.59±22.82 | 0.66 |  |
|  | IL-10 (pg/mL) | 3.00±1.37 | 2.66±1.02 | 0.44 |  |
|  | IL-17 (pg/mL) | 4.18±1.72 | 5.03±3.42 | 0.55 |  |
|  | TNF-α (pg/mL) | 2.47±0.65 | 3.27±3.36 | 0.56 |  |

ǂContinuous variables were compared using Student *t* test, and categorical variables were compared using Chi-square test or Fisher’s exact test.
