# Supplementary figures and images for: Genomic epidemiology and antimicrobial susceptibility of clinical Mycoplasma pneumoniae isolates in Shanghai, 2023–2024
Source: Front Cell Infect Microbiol. 2026 Jul 1;16:1747629. doi: 10.3389/fcimb.2026.1747629 (PMC13368468; doi:10.3389/fcimb.2026.1747629)

A

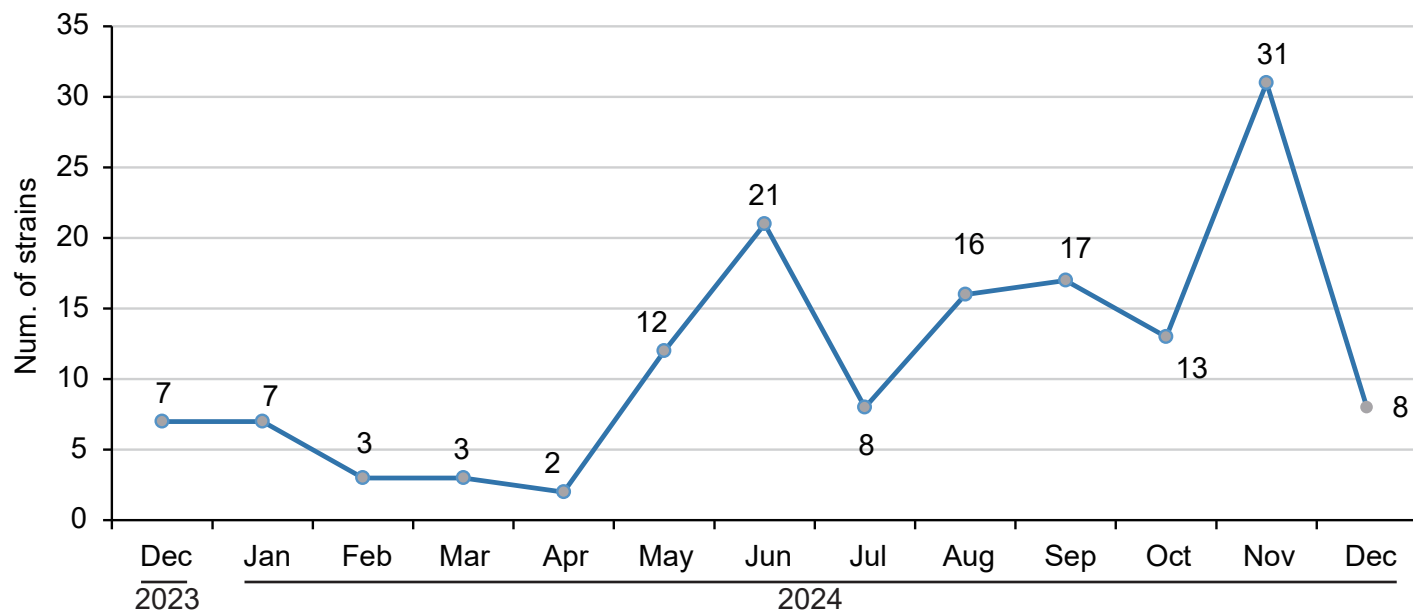

B

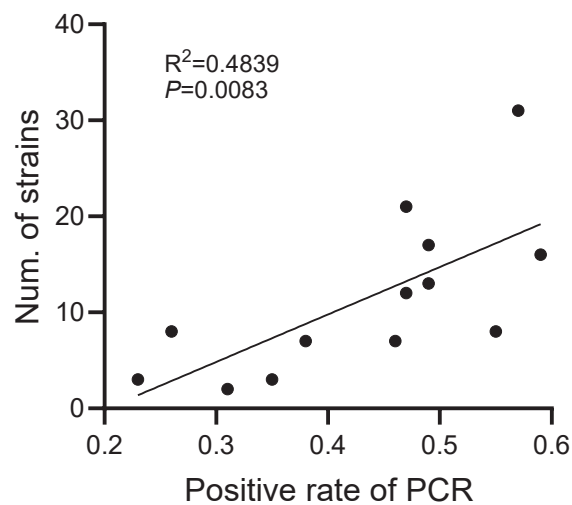

Supplement: Supplementary file 2 [file Image1.pdf]

Distribution of Sequence Types by Region and Time Period (1940-2024)

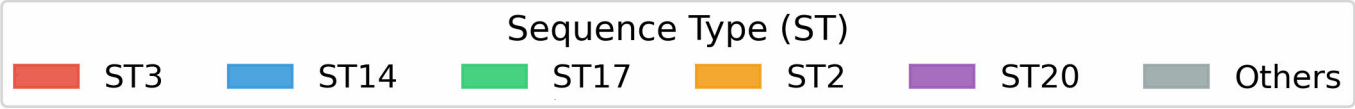

Asia

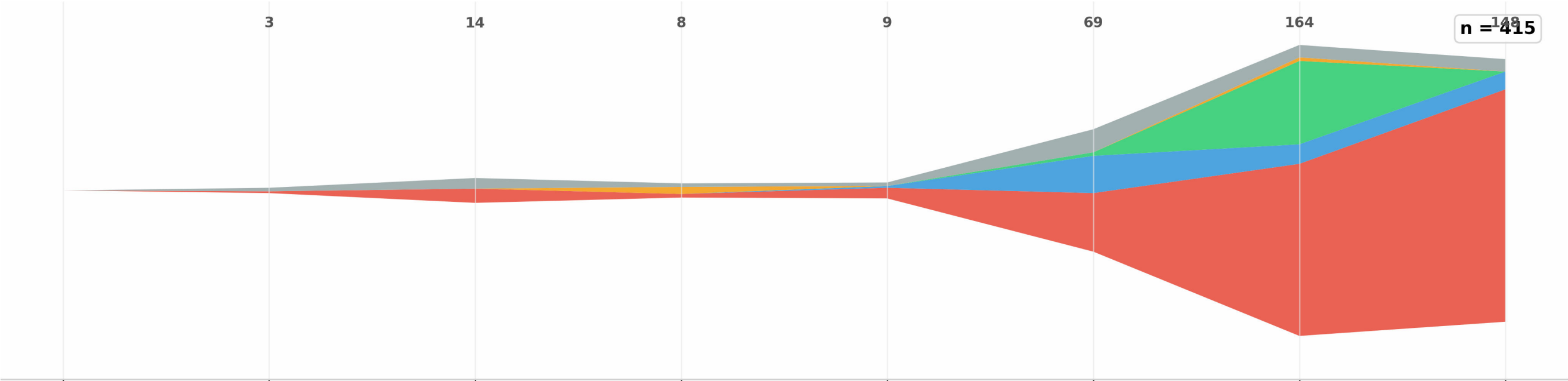

Europe

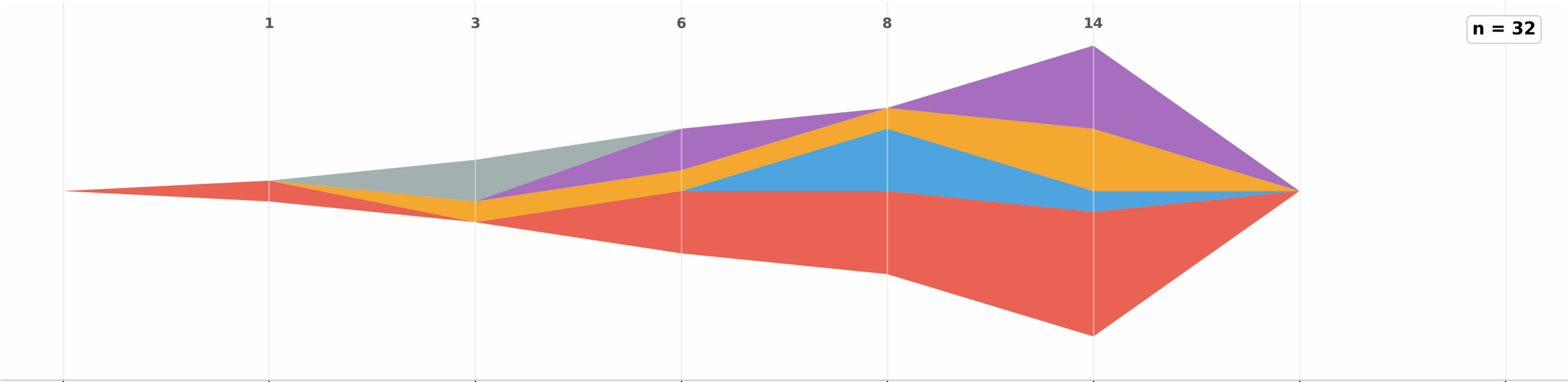

America

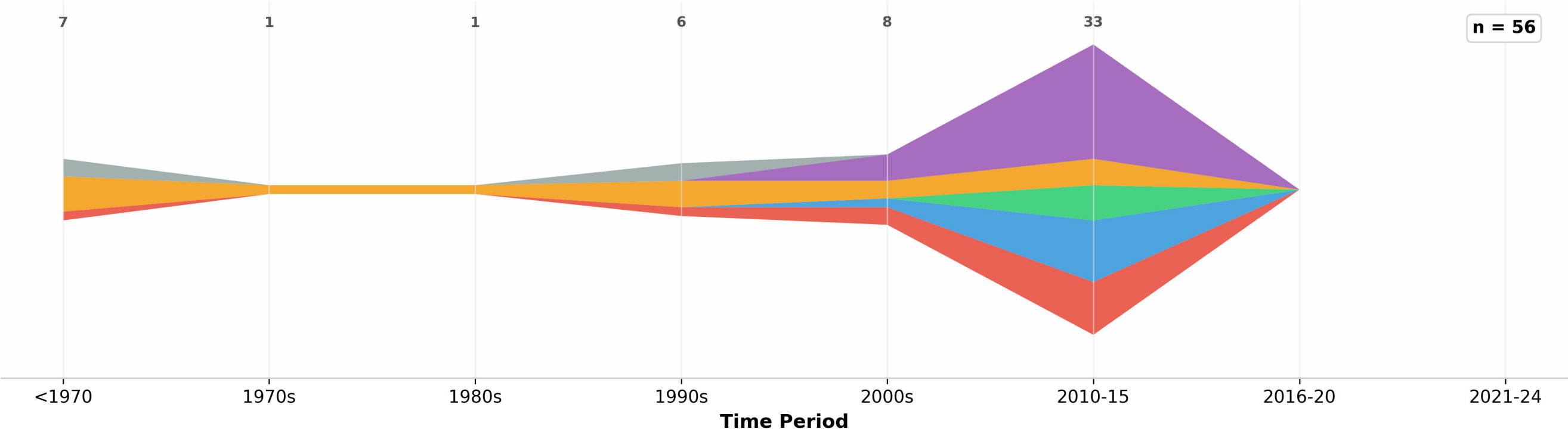

Supplement: Supplementary file 3 [file Image2.pdf]

A

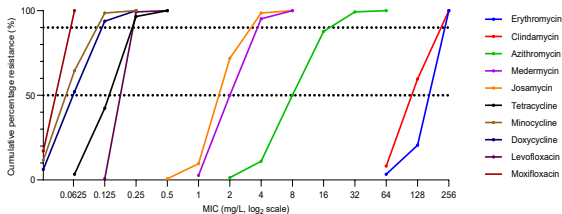

B

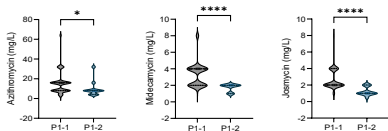

C

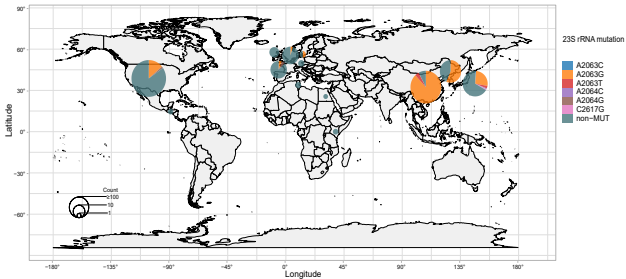

Supplement: Supplementary file 4 [file Image3.pdf]
